# Global trends in anal cancer incidence and mortality

**DOI:** 10.1097/CEJ.0000000000000842

**Published:** 2023-11-27

**Authors:** Silvia Mignozzi, Claudia Santucci, Matteo Malvezzi, Fabio Levi, Carlo La Vecchia, Eva Negri

**Affiliations:** a Department of Clinical Sciences and Community Health, University of Milan, Milan; b Department of Medicine and Surgery, University of Parma, Parma, Italy; c Department of Epidemiology and Health Services Research, Centre for Primary Care and Public Health (Unisanté), University of Lausanne, Lausanne, Switzerland; d Department of Medical and Surgical Sciences, University of Bologna, Bologna, Italy

**Keywords:** age-standardised rates, anal cancer, incidence, joinpoint analysis, mortality, trends

## Abstract

**Objective:**

Anal cancer is a rare disease, affecting more frequently women than men, mainly related to human papillomavirus infection (HPV). Rising incidence and mortality have been reported over the past four decades in different countries.

**Methods:**

To provide an up-to-date overview of recent trends in mortality from anal cancer, we analysed death certification data provided by the WHO in selected countries worldwide over the period from 1994 to 2020. We also analysed incidence derived from Cancer Incidence in Five Continents from 1990 to 2012 for all histologies as well as for anal squamous cell carcinoma (SCC).

**Results:**

The highest age-standardised mortality rates around 2020 were registered in Central and Eastern Europe, such as Slovakia (0.9/100 000 men and 0.40/100 000 women), in the UK (0.24/100 000 men and 0.35/100 000 women), and Denmark (0.33/100 000 for both sexes), while the lowest ones were in the Philippines, Mexico, and Japan, with rates below 0.10/100 000 in both sexes. Upwards trends in mortality were reported in most countries for both sexes. Similarly, incidence patterns were upward or stable in most countries considered for both sexes. In 2008–2012, Germany showed the highest incidence rates (1.65/100 000 men and 2.16/100 000 women).

**Conclusion:**

Attention towards vaccination against HPV, increased awareness of risk factors, mainly related to sexual behaviours and advancements in early diagnosis and management are required to control anal cancer incidence and mortality.

## Introduction

Anal cancer is a rare malignancy, that affects women more frequently than men (GLOBOCAN, 2020). Over 50 000 new cases and 19 000 deaths have been estimated worldwide in 2020. Squamous cell carcinoma (SCC) is the most common anal cancer histology. Other very rare neoplasms of the anal canal are adenocarcinomas, melanomas, sarcomas, and neuroendocrine tumours (Roy *et al*., 2017). Similarly to cervical cancer, the major risk factor for anal SCC is human papillomavirus (HPV) (Daling *et al*., 2004; Goodman *et al*., 2010). Over the last four decades, an increase in anal cancer incidence has been reported in different countries (Islami *et al*., 2017; Kang *et al*., 2018; Deshmukh *et al*., 2020).

In the present paper, we provided an overview of recent mortality trends from anal cancer in European countries and other selected areas of the world up to 2020. We also analysed incidence trends for anal cancer and anal SCC histology for the most populous countries with available data.

## Materials and methods

### Mortality data source and analysis

We extracted official numbers of deaths due to anal cancer in the considered countries from Europe, Australasia, North America, and Latin America from the WHO database (World Health Organization, 2023). The countries have been selected according to population size (for European ones over 5 000 000 inhabitants while for the other countries worldwide over 20 000 000), data coverage (≥90%), and data quality (good-high or good-medium) as declared by the WHO (Anon World Health Organization, 2020). We also analysed the 14 countries that were EU member states before 2004, defined as the EU-14. We could not define the European Union including the 27 member states due to inconsistencies of data in Poland and other central and Eastern European countries.

For each country considered, we analysed numbers of certified deaths since the introduction of the 10th Revision of the International Classification of Diseases up to the most recent available year for each country considered using the code for anal cancer: C21. The calendar period considered was from 1994 to 2020. The EU-14 was built considering calendar years from 2000 to 2018.

Resident populations were extracted from the WHO database for the European and Australasian countries, while from the United Nations (UN) database for the American ones. When data were missing, we used data from EUROSTAT or the UN population division databases.

For each country, sex, and calendar year we computed the age-standardised mortality rates (ASMRs) per 100 000 person-years, using the world standard population, and the related 95% confidence intervals at all ages and at truncated age groups 35–64 and 65 and over. We calculated the ASMRs for the 2005–2009 and 2015–2019 quinquenniums with the corresponding percent change in rates.

In addition, we selected a subset of 24 countries according to the number of deaths (i.e. with more than 10 deaths during the two periods), plus the EU-14. We performed a joinpoint regression analysis on mortality data from this subset of countries. We identified the ‘joinpoint(s)’, where there is a change of the temporal trend’s angular coefficient (on a log scale), with a maximum number of joinpoints of 4. Finally, we reported the annual percent change for each identified linear segment and the weighted average annual percent change (AAPC) over the entire study period.

### Incidence data and analysis

We retrieved incidence data for anal cancer and corresponding population data from the IARC’s Cancer Incidence in Five Continents database (Bray *et al*., 2017), which contains high-quality cancer incidence data provided by national and subnational population-based cancer registries from 1990 to 2012. We considered countries included for mortality that also provided incidence data. For countries with more than one cancer registry, we aggregated data and restricted analyses to the longest common calendar period between registries to ensure the highest geographic coverage. For each country and sex, we derived annual age-adjusted incidence rates for all anal cancer histologies and SCC. Moreover, we reported the percentage of squamous cell cancers during the whole period. We used 3-year moving averages to plot the age-standardised incidence rates.

No ethics committee approval was necessary since we only considered public data. Statistical analyses were performed using the software R version 4.2.0 (R Development Core Team, 2022), SAS version 9.4 (SAS Institute Inc., Cary, NC, USA), and Joinpoint Regression Program version 4.9.1 (Statistical Methodology and Applications Branch, Surveillance Research Program, National Cancer Institute).

## Results

Table 1 gives the ASMRs from anal cancer per 100 000 person-years and the average annual deaths in 2005–2009 and 2015–2019, along with the corresponding percentage change in rates in selected countries worldwide for both sexes. Male ASMRs in 2005–09 ranged between 0.05/100 000 in the Philippines and Mexico and 0.68/100 000 in Slovakia. During the period 2015–2019, the lowest ASMRs were in Mexico and the Philippines (0.05/100 000 and 0.07/100 000, respectively) while the highest was in Slovakia (0.90/100 000), followed by the Czech Republic (0.61/100 000) and Romania (0.42/100 000). Female ASMRs during 2005–2009 varied between 0.04/100 000 in the Philippines and 0.26/100 000 in France. In 2015–2019, these ranged between 0.06/100 000 in the Philippines and Mexico and 0.40/100 000 in Slovakia. Several countries showed higher rates than 0.30/100 000, including Switzerland (0.32/100 000), Denmark (0.33/100 000), the UK (0.35/100 000), and the Czech Republic (0.38/100 000).

**Table 1:** Age-standardised mortality rates in selected European countries, Canada, USA, Japan, and Australia from anal cancers per 100 000 person-years at all ages and average number of annual deaths during 2005–2009 and 2015–2019 (unless indicated in parenthesis), along with the corresponding change in rates (%), according to sex

	Men					Women				
	Annual average deaths 2005–2009	ASMR 2005–2009	Annual average deaths 2015–19	ASMR 2015–2019	% change	Annual average deaths 2005–2009	ASMR 2005–09	Annual average deaths 2015–2019	ASMR 2015–19	% change
Austria	11	0.15	16	0.19	26.7	20	0.19	31	0.25	31.6
Belgium (2018)	11	0.10	17	0.14	40.0	15	0.11	21	0.12	9.1
Bulgaria	12	0.19	13	0.17	-10.5	9	0.11	10	0.11	0.0
Czech Republic	31	0.37	64	0.61	64.9	28	0.23	51	0.38	65.2
Denmark (2018)	7	0.15	20	0.33	120.0	14	0.24	23	0.33	37.5
Finland	6	0.13	9	0.15	15.4	7	0.12	10	0.15	25.0
France (2017)	91	0.17	121	0.19	11.8	203	0.26	268	0.30	15.4
Germany	139	0.18	209	0.23	27.8	213	0.19	322	0.27	42.1
Hungary	9	0.12	11	0.12	0.0	8	0.07	12	0.09	28.6
Italy (2017)	76	0.13	111	0.16	23.1	110	0.12	165	0.17	41.7
Netherlands	17	0.12	31	0.17	41.7	16	0.09	23	0.11	22.2
Norway (2016)	5	0.13	5	0.09	-30.8	10	0.16	10	0.17	6.3
Poland	329	1.20	127	0.37	-69.2	249	0.57	131	0.25	-56.1
Portugal (2018)	13	0.16	18	0.17	6.3	18	0.15	26	0.18	20.0
Romania	64	0.40	77	0.42	5.0	52	0.23	54	0.20	-13.0
Slovakia	24	0.68	41	0.90	32.4	14	0.24	27	0.40	66.7
Spain	40	0.11	66	0.14	27.3	37	0.07	59	0.09	28.6
Sweden (2018)	12	0.13	17	0.16	23.1	25	0.21	33	0.27	28.6
Switzerland	10	0.14	18	0.19	35.7	20	0.24	36	0.32	33.3
UK	104	0.20	155	0.24	20.0	164	0.23	263	0.35	52.2
EU-14 (2018)	428	0.15	644	0.19	26.7	682	0.16	970	0.21	31.3
Argentina	31	0.14	34	0.14	0.0	36	0.13	48	0.14	7.7
Brazil	79	0.10	212	0.18	80.0	160	0.16	343	0.24	50.0
Colombia (2017)	16	0.09	27	0.10	11.1	27	0.12	54	0.16	33.3
Mexico	21	0.05	33	0.05	0.0	24	0.05	42	0.06	20.0
Venezuela (2016)	11	0.11	23	0.17	54.5	20	0.17	48	0.30	76.5
Canada	34	0.13	51	0.15	15.4	43	0.13	84	0.20	53.8
USA	256	0.12	462	0.17	41.7	421	0.16	715	0.22	37.5
Japan	166	0.11	223	0.12	9.1	156	0.07	218	0.08	14.3
Philippines	14	0.05	29	0.07	40.0	12	0.04	28	0.06	50.0
Australia	27	0.16	40	0.17	6.3	34	0.17	54	0.21	23.5

ASMR: age-standardised (world population) mortality rate.

**Fig. 1:**
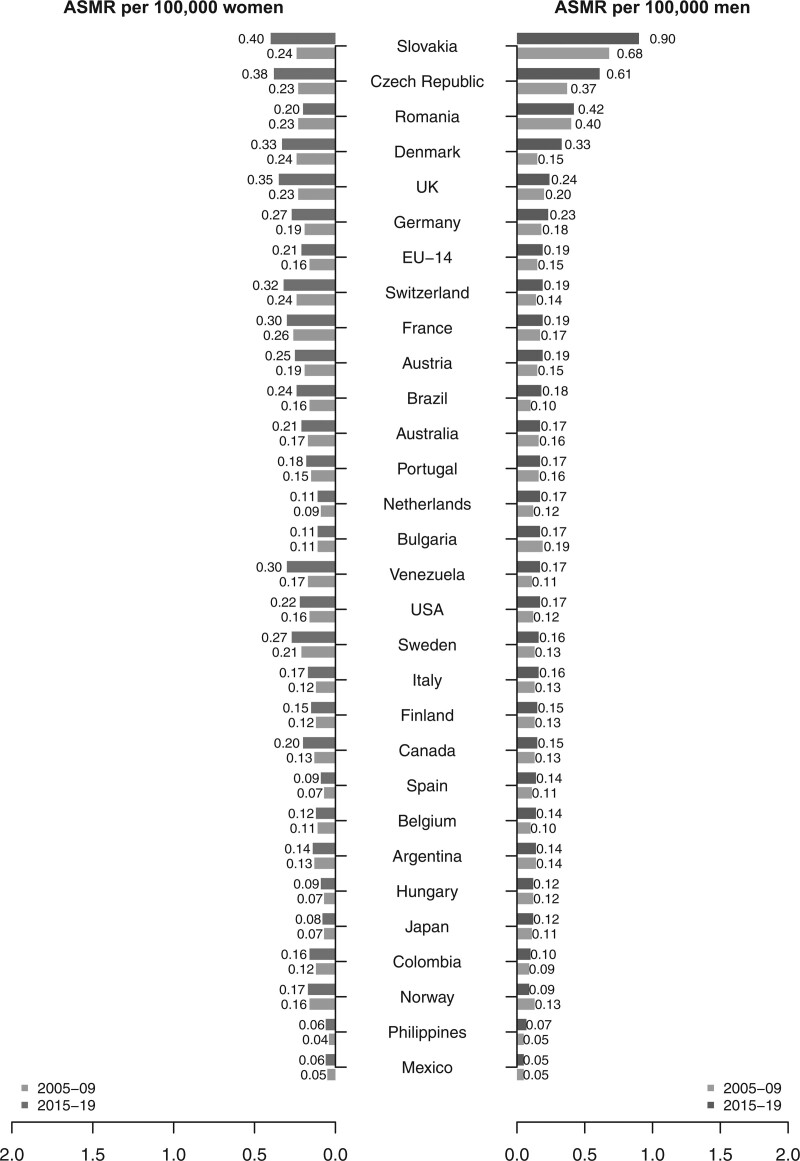
Bar plots of age-standardised (world population) mortality rates per 100 000 persons from anal cancer for the periods 2005–2009 and 2015–2019 in men and women separately in selected countries worldwide.

Figure 1 illustrates the corresponding data in descending order according to ASMRs per 100 000 for men in 2015–2019. Countries from Central and Eastern Europe reported higher ASMRs in both sexes. Additionally, in almost all countries and both sexes the ASMRs increased from 2005–2009 to 2015–2019, except for Bulgarian men (−10.5%), Norwegian men (−30.8%), and Romanian women (−13%; Table 1 and Fig. 1).

Supplementary Tables S1 and S2, Supplemental Digital Content 1, http://links.lww.com/EJCP/A408 report the corresponding figures for men and women aged 35–64 years and over 65 years. ASMR increased in adults aged 35–64 years from 2005–2009 to 2015–2019 in almost all countries considered, with the exception of Romanian women and Portugal, Mexican and Austrian women, and Japanese men who did not report any change in rate between the two periods. Again Slovakia showed the highest mortality rates in both sexes while the Philippines and Mexico reported the lowest ones. In the older age group, most countries considered reported an increase in mortality rates between 2005–2009 and 2015–2019, with some exceptions among males in Hungary (−19.2%), Argentina (−33.3%), and Mexico (−2.9%) and in Romanian women (−3.4%).

**Fig. 2:**
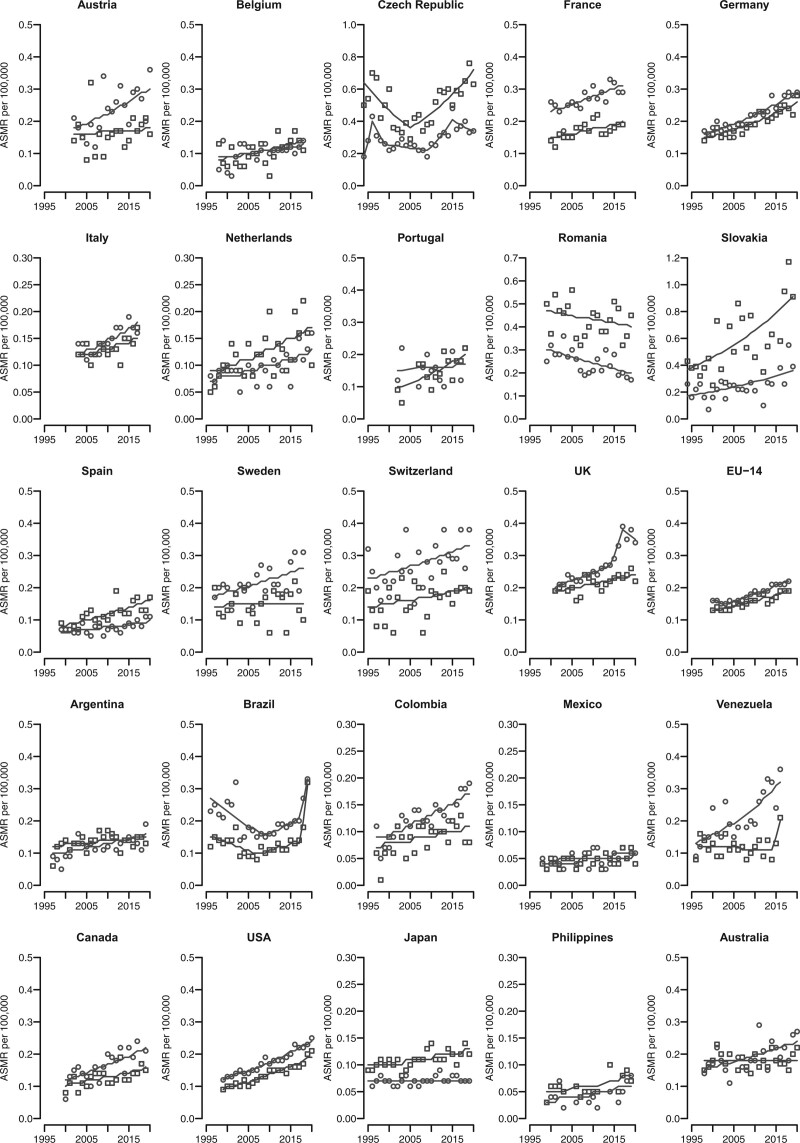
Trends in age-standardised (world population) mortality rates (dots) per 100 000 persons and corresponding joinpoint models (lines) for anal cancer in selected major countries worldwide, among men (black) and women (grey).

Joinpoint analysis for age-standardised death rates in selected countries is shown in Fig. 2 and the corresponding results are given in Supplementary Tables S3a-S3b, Supplemental Digital Content 1, http://links.lww.com/EJCP/A408. Mortality due to anal cancer followed unfavourable trends for most countries considered and both sexes. Male mortality trends increased, with AAPCs from 1.2–1.3 respectively in Japan and UK to 4.7 in Portugal. Austrian, Romanian, Swedish, Argentinian, and Australian men reported approximately stable trends. Similarly, females showed upward trends with AAPCs varying between 1.6 in Switzerland and 3.3 in Canada and the USA. For Portuguese, Brazilian, and Japanese women the observed trends were almost stable over time.

Table 2 gives age-standardised incidence rates, annual average incidence cases of anal cancer during 2008–2012, and the percentage of SCC histology in selected countries worldwide according to sex. For all considered countries, the incidence rates were higher in women than men, except in Slovakia, Spain, Japan and the Philippines. For both sexes, the highest incidence rate was recorded in Germany (1.65/100 000 men and 2.16/100 000 women) while the lowest one was in the Philippines (0.25/100 000 men and 0.18/100 000 women). SCC percentage varied largely in both sexes, with values over 50% in most countries, except in the Philippines (6.7% for men and 11.1% for women), Japan (15.7% in men and 37.8% in women), and Italy (42.2% in men).

**Table 2:** Age-standardised (world population) incidence rate and annual average incidence cases from anal cancer during 2008–2012, along with the percentage of squamous cell carcinoma histology in selected countries worldwide, according to sex

Country	Men			Women		
	Annual average incidence cases	Incidence rate	% squamous	Annual average incidence cases	Incidence rate	% squamous
Austria	52	0.67	63.8	102	1.11	75.4
Bulgaria	20	0.32	52.0	31	0.41	64.9
Czech Republic	47	0.55	50.8	83	0.82	75.5
Denmark	38	0.82	85.9	85	1.73	90.1
France	42	0.72	88.8	113	1.59	92.5
Germany	38	1.65	74.1	60	2.16	77.5
Italy	36	0.58	42.2	62	0.86	68.4
Netherlands	82	0.60	85.8	99	0.68	85.3
Norway	24	0.58	93.2	45	1.03	90.7
Poland	2	0.20	66.7	4	0.33	80.0
Slovakia	22	0.60	32.3	24	0.51	66.7
Spain	42	0.54	48.4	36	0.41	57.9
Switzerland	19	0.84	63.4	52	2.08	90.0
UK	387	0.82	73.0	664	1.26	82.7
Brazil	2	0.43	75.0	8	1.05	80.5
Colombia	9	0.94	51.1	25	1.93	73.0
Australia	150	0.90	66.7	221	1.24	80.6
Canada	175	0.85	64.0	289	1.28	79.5
USA	219	1.09	80.3	323	1.37	88.2
Japan	42	0.32	15.7	48	0.26	37.8
Philippines	6	0.25	6.7	5	0.18	11.1

**Fig. 3:**
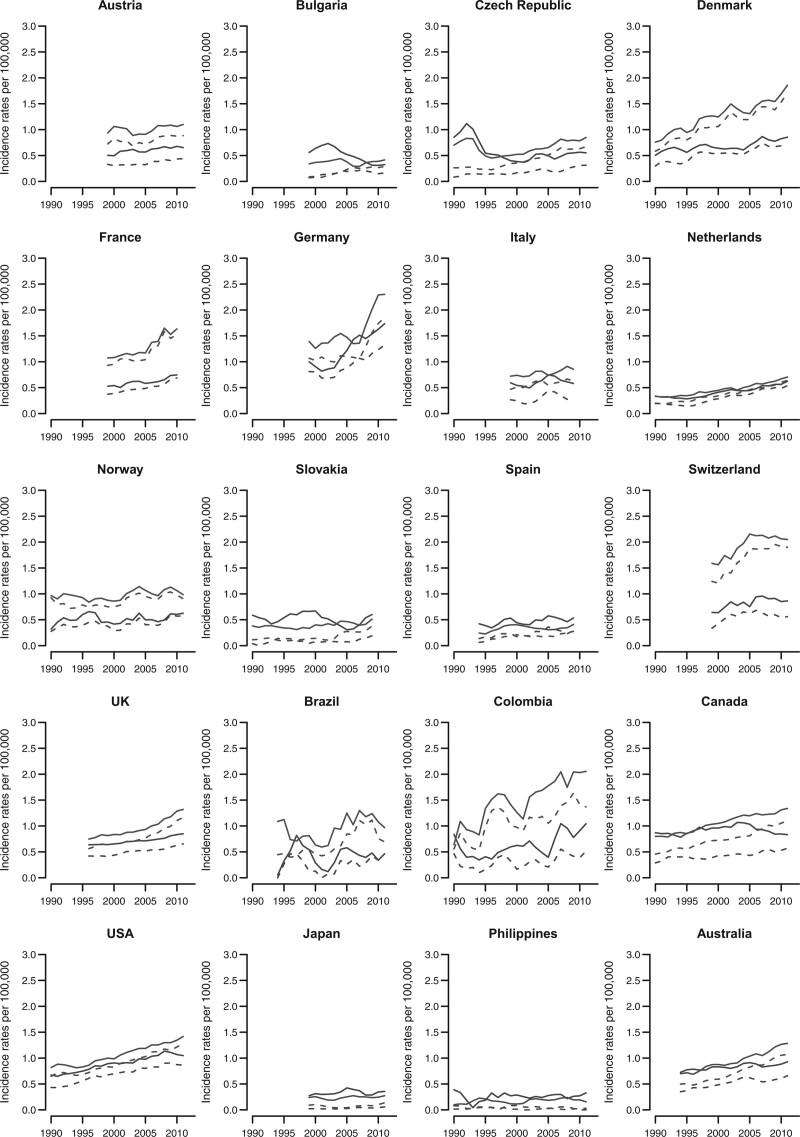
Trends in age-standardised (world population) incidence rates per 100 000 persons for all histologies (line) as well as for SCC (dashed line) anal cancer in selected major countries worldwide, among men (black) and women (grey).

Figure 3 shows the incidence trends for anal cancer in selected countries worldwide according to sex for all histologies (line) as well as for SCC (dashed line). Most countries considered showed unfavourable incidence patterns over time for both sexes, with the exception of Italy, Spain, Japan, and the Philippines which showed moderate growths over time. Female incidence rates from all histologies of anal cancer remained higher than male ones in almost all countries analysed, except Spain, Japan, and the Philippines where males reported higher incidence rates and, in the Netherlands, where there were no noticeable differences.

## Discussion

This is a comprehensive and updated analysis of worldwide geographic patterns and temporal trends in anal cancer incidence and mortality, using the official death certifications collected from the WHO. To reduce variability, we only included countries with WHO death certification data with over 90% coverage, with good data quality (Anon World Health Organization, 2020), and with a resident population of over 5 million. Incidence data provided by Cancer Incidence in Five Continents often refer to subnational registries covering only a limited proportion of the national population and are only updated to 2012 (Bray *et al*., 2017). However, these are the best available data for the evaluation of cancer subsites across different countries.

Anal cancer is a rare tumour often anticipated by anal intra-epithelial neoplasia (AIN) (Palefsky, 1998; Duncan *et al*., 2015). In the present analysis, we observed age-standardised incidence (less than 2.2/100 000 person-years) and mortality (less than 1/100 000 person-years) rates across selected countries worldwide. In most countries, the time trends were unfavourable. Our results are in line with other studies conducted on anal cancer incidence and mortality trends as well as the histology distribution of this neoplasm (Islami *et al*., 2017; Deshmukh *et al*., 2020; Welten *et al*., 2021). With reference to histology, most anal cancers were SCC. However, in countries with very low incidence, such as Japan and the Philippines, SCC accounted only for a minority of anal cancers. This suggests the presence of a baseline of other histology anal cancers, such as sarcomas, adenocarcinomas, melanomas, and neuroendocrine tumors unrelated to HPV. HPV-related SCC anal cancers appear therefore to largely explain the geographic variation.

More than 80% of anal SCC (Patel *et al*., 2020; Welten *et al*., 2021), in fact, are associated with HPV infection. The high mortality rates observed in Eastern European countries can be explained by the higher prevalence of HPV which was around 21% among women as compared to the worldwide one, estimated at around 11% (Forman *et al*., 2012). As for cervical and vulvar (Li *et al*., 2023) cancer, the most common oncogenic serotypes associated with HPV are 16 and 18 (Clark *et al*., 2004; Palefsky *et al*., 2011; Welten *et al*., 2021). Cervical and anal HPV infections often were observed in the same patients. Thus, HPV transmission between the anus and cervix is possible (Goodman *et al*., 2010).

Starting from 2006 in the USA and 2007 in the EU, HPV vaccination campaigns have been introduced (Bruni *et al*., 2021). This prevents both HPV infection and AIN, thus anal cancer (Palefsky *et al*., 2011; Islami *et al*., 2017; Welten *et al*., 2021). Among countries with the highest HPV vaccination coverage, there are those from Northern Europe and Australia which reached almost 70% among females aged 15–19 years (Bruni *et al*., 2021). However, since the most frequent age target for immunisation campaigns is 12 years and the vaccination was introduced in 2006, it is too early to see the substantial effects on anal cancer incidence rates in the adult and elderly population (Bruni *et al*., 2021). Several studies documented the presence of HPV DNA on hands and fingers, which could therefore act as a conduit (Winer *et al*., 2010; Widdice *et al*., 2013; Moscicki *et al*., 2014). A history of genital warts seems to be an important risk factor for both sexes (Daling *et al*., 1987). Men who have had genital warts appear to have a 7-fold risk of developing anal cancer as compared to men who have never had them (Daling *et al*., 2004).

HPV and consequently anal cancer are related to sexual habits, including anal intercourse, age at first intercourse, number of sexual partners, and non-use of condoms (Ryan *et al*., 2000; Daling *et al*., 2004; van der Zee *et al*., 2013; Valvo *et al*., 2019) (Daling *et al*., 1982). The risk of anal cancer is associated with sexual orientation; among men, those who had non-exclusively heterosexual relationships, have a higher risk. Men reporting more than 15 sexual partners have a high risk of anal cancer (with the risk rising for the non-exclusively heterosexual) (Daling *et al*., 2004) (Uronis and Bendell, 2007). Analogously for women, the risk increases directly with the number of male sexual partners. The regular use of condoms during sexual intercourse is inversely related to HPV infection (Islami *et al*., 2017): a 2-fold higher risk of HPV among men who do not use condoms as compared to those who use condoms with unstable partners was estimated (Pierce Campbell *et al*., 2013). A low age at first sexual intercourse, a high number of male sexual partners, and anal touches may postpone HPV clearance (Islami *et al*., 2017).

Over recent calendar periods in most countries, the age at first intercourse declined and the number of sexual partners increased (Wellings *et al*., 2006; Islami *et al*., 2017). The frequency of receptive anal intercourse in both men and women also increased in the US (Herbenick *et al*., 2010; Islami *et al*., 2017). These may contribute to anal cancer time trends. Along this line, we observed lower incidence and mortality rates in Asian countries, particularly in women, where the number of women’s sexual partners and the prevalence of cervical HPV infection were lower than in Europe or America (Islami *et al*., 2017).

Anal cancer and HPV infections are common in HIV-infected subjects (Bower *et al*., 2004; Islami *et al*., 2017). Immunosuppression caused by HIV remains associated with an increased risk of anal SCC, as it may expedite the progression of AIN into anal cancer (Chiao *et al*., 2005). In particular, the prevalence of HPV and AIN was very high among HIV-positive homosexual men (Palefsky *et al*., 1998; Goldstone *et al*., 2001), but a high prevalence was also observed in HIV-positive women (Palefsky, 1998; Islami *et al*., 2017). Several studies investigated the relationship between the rising incidence rate of anal cancer and the introduction of Highly Active Antiretroviral Therapy (HAART) (Bower *et al*., 2004; Chiao *et al*., 2005; Duncan *et al*., 2015; Jin *et al*., 2019). The unfavourable trend in incidence and mortality anal cancer rates is therefore related to HAART, since prolonged survival in HIV-infected individuals resulted in an increased risk for this neoplasm (Bower *et al*., 2004; Chiao *et al*., 2005; Islami *et al*., 2017). Indeed, the prolonged immunosuppressed status may give high-grade squamous intra-epithelial lesions time to develop into anal cancer (Bower *et al*., 2004; Chiao *et al*., 2005).

Anal cancer, like other viral-related neoplasms, was associated with immunosuppression following transplantation or due to other diseases and also to the use of corticosteroids (Daling *et al*., 2004; Islami *et al*., 2017). Post-transplant immunosuppression is a risk factor for viral-related cancers (Ryan *et al*., 2000). From 2006 to 2011, there was an increase in the number of transplants (White *et al*., 2014). This may have contributed to the observed increase in incidence and mortality rates. Transplantation results have improved over the years, but the consequences of immunosuppressive therapy remain (Engels *et al*., 2011).

Smoking is an additional risk factor for anal cancer in both sexes. The association appears to be weaker in women over 60 years (Daling *et al*., 2004). This relationship could be explained by the effect of smoking on the delayed elimination of anal HPV (Shvetsov *et al*., 2009; Islami *et al*., 2017) besides carcinogenic chemicals in tobacco smoking. It is also likely the association is partly due to the residual confounding by sexual factors (Frisch, 2002; Valvo *et al*., 2019). Smoking trends in both Europe and America have been favourable (Anon Eurostat, 2023; Anon American Lung Association, 2023; Marcon *et al*., 2018), in contrast to the unfavourable incidence and mortality observed in the present study. The smoking prevalence in Europe during 2017–2018 registered the highest rates in countries from the central and eastern zone, that is, about 37% in Bulgaria and 34% in Romania (Gallus *et al*., 2021). This could also partly explain the high incidence and mortality rates observed in the present study. Moreover, smoking prevalence was comparably high in France where we found the highest percentages of anal SCC histology for both sexes.

### Conclusion

There are currently no uniform indications for anal cancer screening or early diagnosis, although digital anal rectal examinations and high-resolution anoscopy can detect the early stages of the disease (Nyitray *et al*., 2020; Aninye *et al*., 2021).

Attention towards vaccination against HPV, as well as improving the awareness of sexual-related risk factors, together with progress in diagnosis and management are priority measures for anal cancer control worldwide.

In conclusion, we observed unfavourable trends for both anal cancer incidence and mortality, in particular among Central and Eastern European countries and the UK, where unfavuorable ASMRs are also predicted (Smittenaar *et al*., 2016). The increases in incidence could be related to the unfavourable pattern of recognised risk factors as well as the high prevalence of these in certain regions (UNAIDS, 2022; Wellings *et al*., 2006; Forman *et al*., 2012; White *et al*., 2014; Islami *et al*., 2017; Gallus *et al*., 2021). The increases in mortality rate are also due to the fact that this neoplasm is often diagnosed at an advanced stage, especially over 50 (Deshmukh *et al*., 2020). In addition, limited advancements in diagnosis and treatment have been registered over the recent calendar years (Symer and Yeo, 2018).

## Acknowledgements

This work was supported by the Italian Association for Cancer Research Foundation (AIRC Foundation, project N. 22987) and by EU funding within the NextGenerationEU-MUR PNRR Extended Partnership initiative on Emerging Infectious Diseases (Project no. PE00000007, INF-ACT). The funding sources had no role in the design and conduct of the study; collection, management, analysis, and interpretation of the data; preparation, review, or approval of the manuscript; and the decision to submit the manuscript for publication.

The data that support the findings of this study are openly available in WHO database at https://platform.who.int/mortality/themes/theme-details/topics/topic-details/MDB/malignant-neoplasms and in IARC’s Cancer Incidence in Five Continents (CI5) database at https://ci5.iarc.fr.

### Conflicts of interest

There are no conflicts of interest.

## Supplementary Material
