# Supplementary material for: Global trends in anal cancer incidence and mortality
Source: Eur J Cancer Prev. 2023 Nov 27;33(2):77–86. doi: 10.1097/CEJ.0000000000000842 (PMC10833181; doi:10.1097/CEJ.0000000000000842)
Supplement: Supplementary file 1 [file ejcp-33-077-s001.pdf]

SUPPLEMENTARY MATERIAL

Title: Global trends in anal cancer incidence and mortality
Journal: European Journal of Cancer Prevention
Authors: Silvia Mignozzi, Claudia Santucci, Matteo Malvezzi, Fabio Levi, Carlo La Vecchia, Eva Negri
Corresponding author: Prof. Carlo La Vecchia, MD. Department of Clinical Sciences and Community Health, University of Milan, Italy. E-mail: carlo.lavecchia@unimi.it

Table of contents

Supplementary Table S1. Age-standardized mortality rates in selected European countries, Canada, USA, Japan, and Australia from anal cancers per 100,000 person-years at 35-64 years and average number of annual deaths during 2005-09 and 2015-19 (unless indicated in parenthesis), along with the corresponding change in rates (%), according to sex. 2

Supplementary Table S2. Age-standardized mortality rates in selected European countries, Canada, USA, Japan, and Australia from anal cancers per 100,000 person-years at 65 or more years and average number of annual deaths during 2005-09 and 2015-19 (unless indicated in parenthesis), along with the corresponding change in rates (%), according to sex. 3

Supplementary Table S3a. Joinpoint analysis for anal cancer for all ages by country, men. 4

Supplementary Table S3b. Joinpoint analysis for anal cancer for all ages by country, women. 5

Supplementary Table S1. Age-standardized mortality rates in selected European countries, Canada, USA, Japan, and Australia from anal cancers per 100,000 person-years at 35-64 years and average number of annual deaths during 2005-09 and 2015-19 (unless indicated in parenthesis), along with the corresponding change in rates (%), according to sex.

	Men					Women				
	**Annual average deaths 2005-09**	**ASMR 2005-09**	**Annual average deaths 2015-19**	**ASMR 2015-19**	**% change**	**Annual average deaths 2005-09**	**ASMR 2005-09**	**Annual average deaths 2015-19**	**ASMR 2015-19**	**% change**
Austria	4	-	6	0.27	-	6	0.31	7	0.31	0.0
Belgium (2018)	3	-	5	0.18	-	3	-	4	-	-
Bulgaria	5	0.30	4	-	-	4	-	3	-	-
Czech Republic	12	0.49	20	0.81	65.3	8	0.30	15	0.61	103.3
Denmark (2018)	4	-	7	0.52	-	5	0.40	7	0.54	35.0
Finland	3	-	2	-	-	2	-	4	-	-
France (2017)	38	0.29	44	0.30	3.4	59	0.44	71	0.46	4.5
Germany	57	0.31	79	0.39	25.8	55	0.30	92	0.44	46.7
Hungary	3	-	4	-	-	2	-	3	-	-
Italy (2017)	23	0.18	35	0.24	33.3	22	0.17	48	0.31	82.4
Netherlands	9	0.22	10	0.26	18.2	6	0.15	7	0.16	6.7
Norway (2016)	2	-	2	-	-	2	-	1	-	-
Portugal (2018)	6	0.26	6	0.26	0.0	7	0.30	8	0.30	0.0
Romania	24	0.59	29	0.63	6.8	15	0.33	12	0.25	-24.2
Slovakia	9	0.86	14	1.09	26.7	4	-	5	0.44	-
Spain	18	0.20	29	0.26	30.0	10	0.11	17	0.15	36.4
Sweden (2018)	4	-	5	0.23	-	7	0.36	10	0.44	22.2
Switzerland	5	0.26	4	-	-	7	0.40	10	0.52	30.0
United Kingdom	45	0.35	53	0.37	5.7	50	0.38	83	0.57	50.0
EU-14 (2018)	168	0.24	232	0.30	25.0	183	0.26	270	0.34	30.8
Argentina	12	0.20	19	0.27	35.0	14	0.21	20	0.26	23.8
Brazil	43	0.15	103	0.28	86.7	67	0.23	140	0.35	52.2
Colombia (2017)	8	0.13	11	0.14	7.7	11	0.17	18	0.20	17.6
Mexico	9	0.06	17	0.09	50.0	11	0.08	16	0.08	0.0
Venezuela (2016)	5	0.15	9	0.20	33.3	8	0.23	22	0.45	95.7
Canada	15	0.20	22	0.25	25.0	17	0.22	30	0.33	50.0
USA	142	0.23	216	0.31	34.8	197	0.30	282	0.38	26.7
Japan	48	0.16	43	0.16	0.0	23	0.07	26	0.09	28.6
Philippines	8	0.08	17	0.12	50.0	5	0.05	14	0.09	80.0
Australia	9	0.21	11	0.22	4.8	14	0.30	19	0.36	20.0

ASMR: age-standardized (world population) mortality rate.
ASMR was not computed for less than 5 deaths.

Supplementary Table S2. Age-standardized mortality rates in selected European countries, Canada, USA, Japan, and Australia from anal cancers per 100,000 person-years at 65 or more years and average number of annual deaths during 2005-09 and 2015-19 (unless indicated in parenthesis), along with the corresponding change in rates (%), according to sex.

	Men					Women				
	Annual average deaths 2005-09	ASMR 2005-09	Annual average deaths 2015-19	ASMR 2015-19	% change	Annual average deaths 2005-09	ASMR 2005-09	Annual average deaths 2015-19	ASMR 2015-19	% change
Austria	7	1.18	10	1.42	20.3	14	1.29	24	2.14	65.9
Belgium (2018)	7	0.83	11	1.06	27.7	12	0.97	17	1.04	7.2
Bulgaria	7	1.28	9	1.36	6.3	5	0.51	7	0.73	43.1
Czech Republic	19	3.06	44	5.10	66.7	20	1.76	36	2.66	51.1
Denmark (2018)	4	-	13	2.30	-	8	1.59	16	2.30	44.7
Finland	3	-	7	1.37	-	5	0.75	6	0.84	12.0
France (2017)	53	1.13	77	1.26	11.5	144	1.73	197	2.19	26.6
Germany	82	1.15	129	1.52	32.2	156	1.27	229	1.81	42.5
Hungary	6	1.04	6	0.84	-19.2	7	0.58	9	0.73	25.9
Italy (2017)	54	0.98	76	1.11	13.3	88	0.95	117	1.03	8.4
Netherlands	8	0.70	20	1.24	77.1	10	0.58	16	0.85	46.6
Norway (2016)	3	-	3	-	-	8	1.36	9	1.91	40.4
Portugal (2018)	7	0.89	12	1.12	25.8	11	0.79	18	1.10	39.2
Romania	38	2.83	48	3.16	11.7	36	1.78	41	1.72	-3.4
Slovakia	14	5.76	26	7.46	29.5	10	2.00	21	3.53	76.5
Spain	22	0.65	36	0.85	30.8	27	0.45	42	0.60	33.3
Sweden (2018)	8	1.01	12	1.13	11.9	18	1.36	24	1.88	38.2
Switzerland	5	0.86	14	1.80	109.3	13	1.52	25	2.17	42.8
United Kingdom	58	1.22	102	1.67	36.9	113	1.57	178	2.27	44.6
EU-14 (2018)	259	1.00	408	1.26	26.0	495	1.12	697	1.45	29.5
Argentina	18	1.05	14	0.70	-33.3	21	0.79	27	0.81	2.5
Brazil	32	0.62	106	1.30	109.7	90	1.24	197	1.77	42.7
Colombia (2017)	8	0.63	15	0.78	23.8	16	0.99	36	1.42	43.4
Mexico	11	0.35	15	0.34	-2.9	13	0.34	26	0.46	35.3
Venezuela (2016)	6	0.85	14	1.51	77.6	12	1.37	26	2.19	59.9
Canada	19	0.93	29	0.93	0.0	26	0.86	54	1.39	61.6
USA	112	0.64	240	1.00	56.3	223	0.89	430	1.40	57.3
Japan	117	0.89	178	0.97	9.0	133	0.57	191	0.64	12.3
Philippines	4	-	10	0.46	-	5	0.26	13	0.41	57.7
Australia	18	1.35	28	1.48	9.6	20	1.04	34	1.39	33.7

ASMR: age-standardized (world population) mortality rate.
ASMR was not computed for less than 5 deaths.

Supplementary Table S3a. Joinpoint analysis for anal cancer for all ages by country, men.

Country	Years1	APC1	Years2	APC2	Years3	APC3	AAPC
Austria	2002-2020	0.5					0.5
Belgium	1998-2018	2.7*					2.7*
Czech Republic	1994-2005	-5.1*	2005-2020	4.7*			0.4
France	2000-2017	1.6*					1.6*
Germany	1998-2020	2.6*					2.6*
Italy	2003-2017	1.9*					1.9*
Netherlands	1996-2020	3*					3*
Portugal	2002-2018	4.7*					4.7*
Romania	1999-2019	-0.7					-0.7
Slovakia	1994-2019	3.7*					3.7*
Spain	1999-2020	3.4*					3.4*
Sweden	1997-2018	0.2					0.2
Switzerland	1995-2019	1.5					1.5
United Kingdom	2001-2020	1.3*					1.3*
EU-14	2000-2018	2.2*					2.2*
Argentina	1997-2019	0.9					0.9
Brazil	1996-2007	-4*	2007-2017	3.1	2017-2019	51.8*	3.1
Colombia	1997-2019	1.7					1.7
Mexico	1998-2020	1.6					1.6
Venezuela	1996-2014	-0.9	2014-2016	36.8			2.4
Canada	2000-2019	2.2*					2.2*
USA	1999-2020	3.6*					3.6*
Japan	1995-2019	1.2*					1.2*
Philippines	1999-2019	2.9*					2.9*
Australia	1998-2020	0.2					0.2

APC: annual percent change. *Significantly different from 0 ($p<0.05$).

Supplementary Table S3b. Joinpoint analysis for anal cancer for all ages by country, women.

Country	Years1	APC1	Years2	APC2	Years3	APC3	Years4	APC4	Years5	APC5	AAPC
Austria	2002-2020	3.1*									3.1*
Belgium	1998-2018	1.8									1.8
Czech Republic	1994-1996	48*	1996-1999	-13.9	1999-2009	-1.6	2009-2015	10.9*	2015-2020	-3.7	2.4
France	2000-2017	1.8*									1.8*
Germany	1998-2020	2.8*									2.8*
Italy	2003-2017	2.7*									2.7*
Netherlands	1996-2020	2.3*									2.3*
Portugal	2002-2018	0.6									0.6
Romania	1999-2019	-2.1*									-2.1*
Slovakia	1994-2019	3.1*									3.1*
Spain	1999-2020	2.2*									2.2*
Sweden	1997-2018	2*									2*
Switzerland	1995-2019	1.6*									1.6*
United Kingdom	2001-2014	2.1*	2014-2017	12.2	2017-2020	-2.6					2.9*
EU-14	2000-2003	-2.4	2003-2018	2.7*							1.8*
Argentina	1997-2019	2.2*									2.2*
Brazil	1996-2009	-4.2*	2009-2017	3.7	2017-2019	26.8					0.9
Colombia	1997-2019	3.3*									3.3*
Mexico	1998-2020	1.6									1.6
Venezuela	1996-2016	4.6*									4.6*
Canada	2000-2019	3.3*									3.3*
USA	1999-2020	3.3*									3.3*
Japan	1995-2019	0.2									0.2
Philippines	1999-2019	3.2*									3.2*
Australia	1998-2020	2*									2*

APC: annual percent change. *Significantly different from 0 ($p<0.05$)
